# In Vitro and Pre-Clinical Evaluation of Locally Isolated Phages, vB_Pae_SMP1 and vB_Pae_SMP5, Formulated as Hydrogels against Carbapenem-Resistant *Pseudomonas aeruginosa*

**DOI:** 10.3390/v14122760

**Published:** 2022-12-11

**Authors:** Samar S. Mabrouk, Ghada R. Abdellatif, Ahmed S. Abu Zaid, Ramy K. Aziz, Khaled M. Aboshanab

**Affiliations:** 1 Department of Microbiology, Faculty of Pharmacy, Ahram Canadian University (ACU), 6th October City, Giza 12566, Egypt; 2 Department of Microbiology & Immunology, Faculty of Pharmacy, Ain Shams University, Cairo 11566, Egypt; 3 Department of Microbiology and Immunology, Faculty of Pharmacy, Cairo University, Cairo 11562, Egypt; 4 Department of Microbiology and Immunology, Children’s Cancer Hospital Egypt 57357, Cairo 11617, Egypt

**Keywords:** carbapenem-resistant, *Pseudomonas aeruginosa*, bacteriophage, hydrogel, thermal injury model, histopathology

## Abstract

The inadequate therapeutic opportunities associated with carbapenem-resistant *Pseudomonas aeruginosa* (CRPA) clinical isolates impose a search for innovative strategies. Therefore, our study aimed to characterize and evaluate two locally isolated phages formulated in a hydrogel, both in vitro and in vivo, against CRPA clinical isolates. The two phages were characterized by genomic, microscopic, phenotypic characterization, genomic analysis, in vitro and in vivo analysis in a *Pseudomonas aeruginosa*-infected skin thermal injury rat model. The two siphoviruses belong to class Caudovirectes and were named vB_Pae_SMP1 and vB_Pae_SMP5. Each phage had an icosahedral head of 60 ± 5 nm and a flexible, non-contractile tail of 170 ± 5 nm long, while vB_Pae_SMP5 had an additional base plate containing a 35 nm fiber observed at the end of the tail. The hydrogel was prepared by mixing 5% *w*/*v* carboxymethylcellulose (CMC) into the CRPA propagated phage lysate containing phage titer 10^8^ PFU/mL, pH of 7.7, and a spreadability coefficient of 25. The groups were treated with either Phage vB_Pae_SMP1, vB_Pae_SMP5, or a two-phage cocktail hydrogel cellular subepidermal granulation tissues with abundant records of fibroblastic activity and mixed inflammatory cell infiltrates and showed 17.2%, 25.8%, and 22.2% records of dermal mature collagen fibers, respectively. In conclusion, phage vB_Pae_SMP1 or vB_Pae_SMP5, or the two-phage cocktails formulated as hydrogels, were able to manage the infection of CRPA in burn wounds, and promoted healing at the injury site, as evidenced by the histopathological examination, as well as a decrease in animal mortality rate. Therefore, these phage formulae can be considered promising for clinical investigation in humans for the management of CRPA-associated skin infections.

## 1. Introduction

Gram-negative bacterial infections present significant treatment challenges in clinical settings, imposing limited therapeutic options [1,2,3]. Although many Gram-negative bacteria are clinically significant, *Pseudomonas (P.) aeruginosa* is one of the most common healthcare-associated pathogens and is considered a major threat by the Centers for Disease Control [3]. Approximately 51,000 healthcare-associated infections (HAIs) were caused by the opportunistic pathogen *P. aeruginosa* each year in the United States (USA) from 2011 to 2014 [3]. *P. aeruginosa* ranked third among Gram-negative causes of selected HAIs reported to the National Healthcare Safety Network (NHSN) [1,2,3].

*P. aeruginosa* infections are frequently associated with pneumonia, bloodstream, urinary tract, and surgical site infections, as well as significant morbidity and mortality rates. They are often difficult to treat due to their intrinsic resistance to many commonly used antimicrobial drugs. Accordingly, carbapenems have emerged and have been widely used as crucial antimicrobial agents for the clinical treatment of severe *P. aeruginosa* infections. As a result, an increasing problem of carbapenem resistance has been reported, considering that carbapenem antibiotics are thought to be the final line of defense against extremely severe multidrug-resistant infections [3,4,5,6,7].

Carbapenem-resistant *P. aeruginosa* (CRPA) can contribute to an increase in mortality, prolonged hospital stays, and other issues, including rising medical expenditures [8]. In rare situations, the only antibiotic that is still effective is colistin. However, colistin’s therapeutic usage has been constrained by both nephrotoxicity and neurotoxicity [9], and that colistin-resistant strains have also been reported [9,10,11]. With treatment failures emerging in tandem with rising antibiotic resistance worldwide, interest in new treatment options against CRPA infections has been evoked. One of the most popular alternative treatment options in studies is bacteriophage therapy [12,13].

Phage therapy is known as the direct application of lytic phages to a patient, targeting a lysing bacterial pathogen causing a clinically relevant infection [14]. Lytic bacteriophages are used to treat infections such as upper respiratory tract, abscesses, burns, and wound infections [15]. In addition, bacteriophages offer several benefits, including the ability to combat bacterial biofilms and protect the natural microbiota. They are also non-toxic, inexpensive, and easy to obtain, and hence, they are one of the most promising alternative options in the treatment of such infections [16].

Although several studies have been carried out to test the lytic activity of various bacteriophages either alone as phage lysates or as cocktails against clinically relevant pathogens including *P. aeruginosa*, they are mostly limited to in vitro examination, and only a few studies have recently been conducted in vivo [17,18,19,20,21]. In addition, till now no studies were conducted for pharmaceutical preparation and in vivo evaluation of suitable topical preparations containing these biologically active phage lysates. Therefore, in this study, two newly isolated bacteriophages with lytic activity have been evaluated both in vitro and preclinically, either as lysates or as hydrogel formulae, against a CRPA clinical isolate that was recovered from severely burned infected patient and showed extensively resistant (XDR) phenotype.

## 2. Materials and Methods

### 2.1. Clinical Bacterial Isolates: Collection, Identification, and Antimicrobial Susceptibility Testing

Three *P. aeruginosa* clinical isolates were recovered from discharged unidentified wound exudates of patients who had been admitted to the El-Demerdash Tertiary Care Hospital, Cairo, Egypt. Based on the hospital records, the respective samples were collected from unidentified patients suffering from severe burn infections as a routine checkup for culture and sensitivity. The protocol of this study was approved by the Faculty of Pharmacy Ain Shams University Research Ethics Committee (Number, ACUC-FP-ASU RHDIRB2020110301 REC# 41 in September 2021). Using Bergey’s manual of determinative bacteriology, isolates were identified macroscopically, microscopically, and biochemically [22]. In addition, bacterial identification was confirmed using the VITEK2 automated system [23]. The isolates were also assessed for their pattern of susceptibility to amikacin (AK), aztreonam (AT), ciprofloxacin (CIP), levofloxacin (LEV), imipenem (IMP), and meropenem (MRP) using the Kirby-Bauer disk diffusion according to CLSI guidelines [24]. The isolates exhibiting the multidrug-resistant (MDR) and XDR phenotypes were defined using previously reported international standard criteria [25]. The XDR isolates that showed a resistance pattern to any of the carbapenems tested were potentially identified as carbapenem-resistant and were chosen to test their MIC against imipenem using the CLSI broth microdilution method according to CLSI guidelines, 2021 [24].

### 2.2. Phenotypic and Genotypic Detection of Carbapenemase Producers (CPs)

The carbapenemase producer isolates were detected using different phenotypic tests, including the modified carbapenem inactivation method (mCIM), combined disk test (CDT), and blue-Carba test (BCT). The genomic DNA from the phenotypically confirmed XDR CP isolates was isolated using the genomic DNA purification kit (Thermo Fisher Scientific, Massachusetts, USA) and was utilized as a template for PCR to test for the five major carbapenemase genes, including *bla*_KPC_, gene coding for *Klebsiella pneumoniae* carbapenemases (KPC); *bla*_NDM_, a gene coded for New Delhi metallo-β-lactamase (NDM); imipenem-resistant *Pseudomonas*-type carbapenemases (IMP); *bla*_VIM_, a gene coded for Verona integron-encoded metallo-β-lactamase (VIM); and *bla*_OXA-48,_ oxacillinase (OXA-48-like) types as previously described [26].

### 2.3. Bacteriophage Recovery from Sewage Samples

#### 2.3.1. Isolation of *P. aeruginosa*-Specific Bacteriophages

The samples were collected from the sewage sources of the specialized Ain Shams University Hospitals, in Cairo, Egypt. The sewage sources of the respective specimens were selected based on the probability of incorporating *P. aeruginosa* and, consequently, bacteriophages specific against it [27]. All samples were kept at 4 °C until processing and were handled based on their apparent clarity [19]. Samples that were visibly clear were used as-is, while Wetted cotton and filter paper were used to thoroughly filter turbid samples [19]. For further processing, only the clear supernatant was preserved. For the isolation of bacteriophages, CRPA isolates served as the bacterial hosts. A loopful of each bacterial isolate cultivated on a nutrient agar plate was inoculated into tryptic soy broth (TSB) and incubated for 6–7 h in a 37 °C, 180 rpm shaking water bath. The suspension of each bacterial isolate was utilized when heavily turbid, with an optical density identical to a bacterial count of 10^9^ CFU/mL [28]. For isolation of the phage, a double-strength broth (TSB) was prepared, with 50 µL of 1 M Ca and 25 µL of 0.5 M Mg added in particular [29]. A suspension containing the bacterial host inoculum, the environmental sample, and the isolation medium with a proportion of 1:1:10, respectively, was incubated overnight at 28 °C and 180 rpm [30]. The following day, the co-culture was centrifuged for 20 min at 6000 rpm. The supernatant was agitated vigorously for five minutes after the addition of chloroform with a ratio of 1:10 [19]. The suspensions were allowed to separate for 4–6 h at 4 °C, and the supernatant that formed on top of a plug-like sediment was collected and re-centrifuged under the same circumstances. The collected lysates were stored at 4 °C [30].

#### 2.3.2. Screening for Lytic Activity against CRPA in the Acquired Lysates

A spot test was used to qualitatively screen for anti-CRPA bacteriophages in the fresh lysates. The procedure was carried out in accordance with Adams’ original description of the method [31]. The presence of phages active against CRPA was indicated by the presence of clear inhibition spots [31]. The quantitative plaque assay was next performed on the lysates that had positive spot test results using the standard double agar overlay (DAO) method [32]. Plaques were examined and counted the following day. The following equation was used to calculate the phage titer [33]:

Phage titer in plaque-forming unit per ml (PFU/mL) = number of plaques/volume of lysate infected × dilution factor

#### 2.3.3. Phage Propagation

The same isolation procedure was repeated three times, but each time an aliquot of the obtained crude phage lysate was used instead of the starting sewage sample. Essentially, propagation was done frequently to maintain a sizable stock of high-titer phage suspensions [19,34].

### 2.4. Characterization of the Isolated Bacteriophages Showing Lytic Activities against CRPA

#### 2.4.1. Host Range

The host range was determined as previously reported [35]. The selected lysates showing positive spot test results were tested for lytic activity against the remaining three CRPA isolates and some other clinically relevant pathogens, including three *Klebsiella pneumoniae,* six *Acinetobacter baumannii*, and two *Escherichia coli* clinical isolates that were previously collected and identified in our previous study [26].

#### 2.4.2. Morphology of the Isolated Bacteriophages Showing Lytic Activities against CRPA

For microscopical examination, a concentrated phage suspension of each of the chosen lysates was centrifuged twice at 10,000 rpm for 25 min and was then filtered through a sterile syringe filter (0.22 mm). After that, 20-mL samples were prepared as instructed by Kalatzis et al. and examined via a transmission electron microscope (TEM; version JEOL_JEM_1400 Electron Microscope Nieuw-Vennep, Tokyo, Japan) performed at Cairo University Research Park, Faculty of Agriculture, Cairo, Egypt.

#### 2.4.3. Molecular Analysis of the Bacteriophage

The bacteriophage lysate was sequenced in an Illumina MiSeq instrument (Illumina, La Jolla, CA, USA), and the library was prepared by the Nextera XT DNA Library preparation kit (San Diego, CA, USA). The sequence reads were uploaded into the PATRIC BRC [36] website (now renamed to BV-BRC, URL: https://www.bv-brc.org/, accessed on 23 October 2022) and analyzed through the metagenomics binning pipeline, which uses the BV-BRC database for extraction and annotation of both bacterial and viral genomes from sequence reads [37]. Briefly, reads are assembled by the most optimal assembly tool on the BV-BRC server (either MetaSPAdes or MEGAHIT). The produced assembled contigs are further annotated on PATRIC by the default RAST algorithm [38]. The phage genome was reannotated on the RAST server (https://rast.nmpdr.org, accessed on 23 October 2022) by the RASTtk pipeline, following the customized phage annotation pipeline [39]. The annotation of every protein-coding gene was further checked and confirmed by BLASTP searches against the NCBI NR database, filtered for viruses [40] then by PFAM searches in the case of hypothetical proteins. Curated annotations was generated from the consensus of RAST, PATRIC, and BLAST hits. The creation of the circular image and comparison with other reported similar plasmids were performed using the BLAST Ring Image Generator (BRIG) tool v0.95 (https://sourceforge.net/projects/brig/, accessed on 25 October 2022) [41].

### 2.5. Formulation of Bacteriophage-Carboxymethyl Cellulose (CMC) Hydrogel

The hydrogel was prepared by mixing 5% *w*/*v* CMC (El Nasr Pharmaceutical Chemicals Co. (ADWIC), Cairo, Egypt) in the CRPA propagated phage lysate containing phage titer 10^8^ PFU/mL (tested hydrogel). To ensure the formation of a homogeneous hydrogel, CMC was added in portions by sprinkling CMC powder with constant stirring [18]. Another 5% *w*/*v* CMC in sterile distilled water was formulated as a negative control (control hydrogel). The hydrogel was prepared with a pH of 7.7 and a spreadability coefficient of 25 measured, as previously reported [42].

### 2.6. In Vitro Anti-CRPA Activity of the Tested Hydrogels

The anti-CRPA activity of the tested hydrogels was evaluated with the cup-plate method using Mueller–Hinton agar plates. The CRPA isolate was seeded into a sterilized growth medium, which was then transferred aseptically into Mueller-Hinton agar plates to form a double-layer plate [43]. After complete solidification, a sterile cork-borer with a 5 mm diameter was used to make the cups. After that, the prepared cups were filled with the same volume (about 200 μL) of each of the propagated phage lysates alone (positive control), tested hydrogels and control hydrogel (negative-control) separately, and then incubated at 37 °C for 24 h. The antimicrobial activity was determined by the presence or absence of a zone of inhibition around the cups [43].

### 2.7. Preclinical Evaluation of the Formulated Tested Hydrogels

#### 2.7.1. Laboratory Animals

Throughout the experiment, female Wistar rats weighing approximately 110–120 g were used. All animals were housed in open cages and fed an antibiotic-free diet consisting mainly of 20% protein, 6.5% ash, 5% fiber, and 3.5% fat, with free access to water. They were kept on an alternating 12 h light-dark cycle and a constant temperature of 25 °C adjusted by air conditioning. Animals were maintained in accordance with the Care and Use of Laboratory Animals recommendations and ARRIVE guidelines (https://arriveguidelines.org) (accessed on 23 October 2022) after the study was approved by the Faculty of Pharmacy Ain Shams University Ethics Committee Number, ACUC-FP-ASU RHDIRB2020110301 REC# 41.

#### 2.7.2. Thermal Injury Model

The thermal injury model was performed according to Sakr et al. [18] with a minor modification using a rectangular metal bar (2×2 cm and 1 mm thickness) to induce burns in the backs. The examined animal groups were divided into six control and three test groups, five rats each, as follows:

Group I: Control, Burned, non-infected, untreated.
Group II: Control, burned, infected, untreated.
Group III: Control, Burned, infected, treated with vehicle (control hydrogel).
Group IV: Burned, infected, treated with tested hydrogel-1 (phage vB_Pae_SMP1)
Group V: Burned, infected, treated with tested hydrogel-2 (phage P5).
Group VI: Burned, infected, treated with phage cocktail hydrogel (Phage vB_Pae_SMP1 + phage P5).
Group VII: Positive Control, burned, infected, treated with Silver sulfadiazine 1% (Silvirburn^®^, MUP Co., Cairo, Egypt).
Group VIII: Positive Control, burned, infected, treated with Collagenase 0.6 IU (Iruxol ^®^, Abbott Co., Wiesbaden, Germany).
Group IX: Normal Control, intact, non-infected, untreated.

#### 2.7.3. Treatment

The first application of the tested formulae to the burned skin was at 2 h post-infection. On the infected burn, a weight of approximately 1 g of hydrogel was applied topically. The administration of treatment was applied twice daily for 14 days. The survival rate of animals was recorded three days post-infection. Dead animals were eliminated from the groups and were only considered when calculating mortality rates. Before the surviving animals were sacrificed, blood was aseptically withdrawn from the retro-orbital plexus after lidocaine (4%) local anesthesia [44] and analyzed for bacterial counts, as detailed below. The animals were euthanized by cervical dislocation, and the dorsal skin at the site of the wound was also removed immediately after the animals were sacrificed for histopathological examination [18].

#### 2.7.4. Histopathological Examination

For histopathological examination, dorsal skin samples were flushed and fixed in 10% neutral-buffered formalin for 72 h, dehydrated in serial ascending grades of ethanol, cleared in xylene, and then infiltrated with a synthetic paraplast tissue embedding medium [45]. Using a rotatory microtome, 5 μn thick tissue sections were made at the middle zones of various wound samples to show the different skin layers and then stained with hematoxylin and eosin using standard procedures. In a blind manner, tissues were examined for histopathological changes and, additionally, the content of collagen fibers was quantitatively analyzed by Assistant Professor Dr. Mohamed Abdelrazik, Cytology and Histology Department, Veterinary Medicine, Cairo University, using Masson’s trichrome stain. In this process, 6 non-overlapping fields were arbitrarily chosen and scanned from the dermal layers of each sample to determine the relative area percentage of collagen fibers in the Masson’s trichrome-stained sections in accordance with Almukainzi et al. [46]. All standard procedures for sample fixation and staining were carried out as previously reported [47].

### 2.8. Statistical Analysis

To calculate *p*-values and standard deviation, data were analyzed using a one-way ANOVA test using GraphPad Instat-3 software (Graph Pad Software Inc., San Diego, CA, USA). Results were displayed as corresponding average values ± Standard deviation.

## 3. Results

### 3.1. Identification, Antimicrobial Susceptibility, Phenotypic and Genotypic Analysis of the Recovered P. aeruginosa Isolates

The three isolates were identified as *P. aeruginosa* PA1, PA2, and PA3, respectively. As shown in Table 1, the susceptibility pattern demonstrated that all the isolates exhibited resistance to all the tested antimicrobial agents, including imipenem and meropenem; therefore, they were categorized as CRPA isolates. The MIC of the three CRPA isolates against imipenem, as well as the phenotypic tests and the detected carbapenemase genes, are shown in Table 1.

### 3.2. Recovery of Bacteriophages and Screening for the Activity against CRPA

Screening 15 sewage samples showed that only two samples (namely, vB_Pae_SMP1 and P5) had a positive spot test against CRPA and, therefore, were chosen for further study. Plaque assay results showed that all lysates had relatively reproducible, high initial titers (>10^8^ PFU/mL) (Figure S1). The resulting plaques were clear, circular with regular edges, small (2–5 mm), and encircled by halos (Figure S2).

### 3.3. Characterization of the Isolated Bacteriophages Showing Lytic Activities against CRPA

#### 3.3.1. Host Range

The vB_Pae_SMP1 lysate was relatively active against CRPA2 and CRPA3 isolates, and the vB_Pae_SMP5 lysate was active against CRPA1 and CRPA2 isolates (Table 2). The two lysates were chosen with isolate CRPA2 for further studies (Figure S3), while none of the two phage lysates showed lytic activity against any of the remaining 11 clinically relevant Gram-negative pathogens.

#### 3.3.2. Morphology of the Bacteriophages vB_Pae_SMP1 and vB_Pae_SMP5 as Presented by TEM

**Figure 1:**
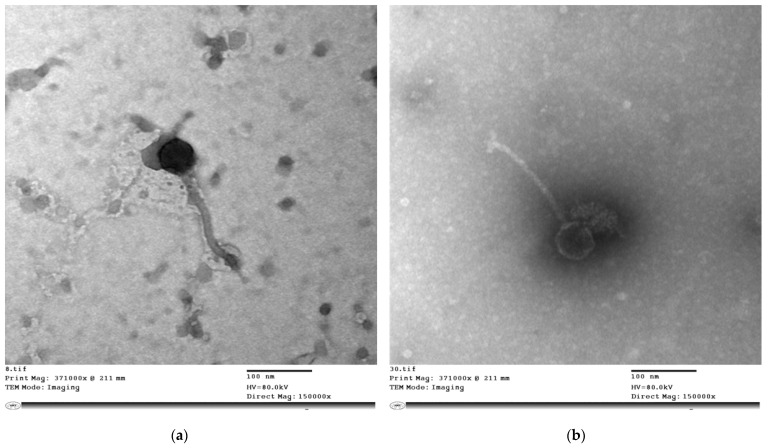
Electron micrograph of bacteriophages; (**a**) vB_Pae_SMP1, 60 ± 5 nm icosahedral head, flexible non-contractile tail 170 ± 5 nm long, (**b**) vB_Pae_SMP5, with an additional base plate containing 35 nm fiber at the end of the tail.

Phage vB_Pae_SMP1 and vB_Pae_SMP5 each has an icosahedral head of 60 ± 5 nm and a flexible non-contractile tail of 170 ± 5 nm long, while vB_Pae_SMP5 has an additional base plate containing a 35 nm fiber observed at the end of the tail (Figure 1a,b). By matching these observations to the data on the “Viral Zone” website along with the guidelines provided by the International Committee on Virus Taxonomy (ICTV), it could be suggested that both phages vB_Pae_SMP1 and vB_Pae_SMP5 fall into the siphoviral morphotype (formerly family Siphoviridae) of the class Caudoviricetes (formerly order Caudovirales); however, Phage vB_Pae_SMP5 has features of members of the genus *Septimatrevirus* (currently a genus under Caudoviricetes, with no assigned family yet).

#### 3.3.3. Phage Lysate Sequencing Results a Complete Genome of Phage vB_Pae_SMP5

**Figure 2:**
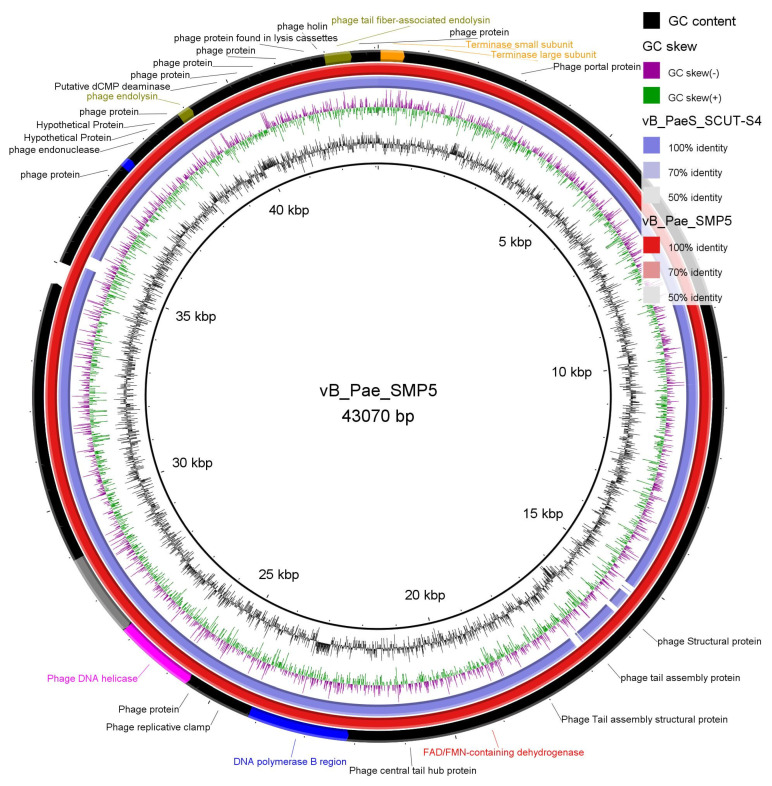
Circular genome map of vB_Pae_SMP5 (Red ring). The BV-BRC accession is 2731619.92. The color coding of genes indicates the functional categories of putative proteins: phage and hypothetical proteins (Black), terminase protein (orange); phage helicase (fuchsia); regulatory protein (purple); phage endolysin (olive); Phage polymerase (blue); exonuclease (Gray); blue ring is the reference phage *Pseudomonas* phage vB_PaeS_SCUT-S4 (NCBI nucleotide accession code MK165658.1). The creation of the circular image and comparison with other reported similar plasmids were performed using the BLAST Ring Image Generator (BRIG) tool v0.95 (https://sourceforge.net/projects/brig/, accessed on 25 October 2022).

High-throughput sequencing of the lysate of phage vB_Pae_SMP5 using the Illumina MiSeq instrument yielded a consensus sequence of 43,070 bp (in one full contig) with 57 protein-coding genes. The full description of putative functions of the resulting open reading frames (ORFs) are shown in Table S1. BLASTN [40] alignment showed that the taxonomy of this phage was Viruses; Duplodnaviria; Heunggongvirae; Uroviricota; Caudoviricetes; Septimatrevirus with an alignment score >200 and 98.0% identity. The genomic and phenotypic characteristics of phage vB_Pae_SMP5 are displayed in Table 3. The genomic analysis of phage vB_Pae_SMP1 is undergoing. The circular genome map of vB_Pae_SMP5 is depicted in Figure 2. The genomic sequence of phage vB_Pae_SMP5 has been deposited in the NIH-funded BV-BRC database, and the BV-BRC accession is 2731619.92 (https://www.bv-brc.org/view/Genome/2731619.92; accessed on 7 December 2022).

### 3.4. In Vitro Anti-CRPA Activity of the Tested Hydrogels

Inhibition zones were observed around the cups containing either phage vB_Pae_SMP1 or vB_Pae_SMP5 lysate (positive control) and around the cups containing either the tested hydrogel of phage vB_Pae_SMP1 or phage vB_Pae_SMP5 (Figure S4), while no inhibition zone was detected around the cups containing the control hydrogel (negative control).

### 3.5. In Vivo Anti-CRPA Activity of the Formulated Tested Hydrogels

#### 3.5.1. Survival Rate

The survival rate of the tested rats for each of the examined groups, calculated until day 14, is tabulated in Table 4.

#### 3.5.2. Histopathological Examination

**Figure 3:**
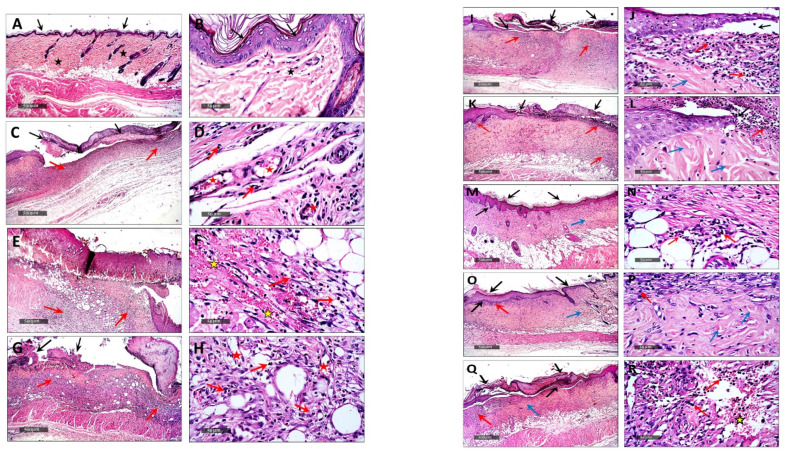
Demonstrating the light microscopic histopathological features of skin layers and wound healing process in different groups; (**A**,**B**): group I, (**C**,**D**): group II, (**E**,**F**): group III, (**G**,**H**): group IV, (**I**,**J**): group V, (**K**,**L**): group VI, (**M**,**N**): group VII (**O**,**P**): group VIII, (**Q**,**R**): group IX. Group codes are shown in Table 4. (H&E stain). Black arrow: Epidermal layer & wound gap. Star: intact skin dermis. Red arrow: Inflammatory cell infiltrates. Red star: congested subcutaneous blood vessels. Blue arrow: Dermal mature collagen fibers. Yellow star: dermal hemorrhagic patches.

**Figure 4:**
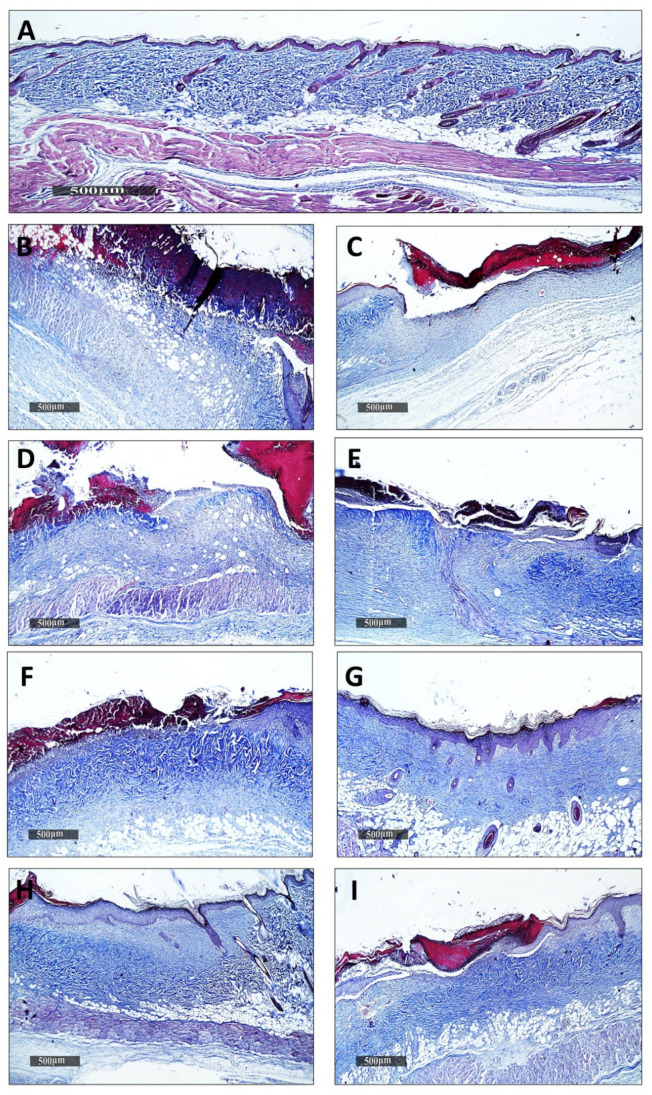
Dermal collagen fibers of different experimental groups; (**A**), dermal collagen fibers of normal health tissue group I; (**B**), group II, (**C**), group III, (**D**): group IV, (**E**), group V, (**F**), group VI; (**G**), group VII; (**H**), group VIII; (**I**), group IX, Group codes are shown in Table 4. (Masson’s Trichome stain).

**Figure 5:**
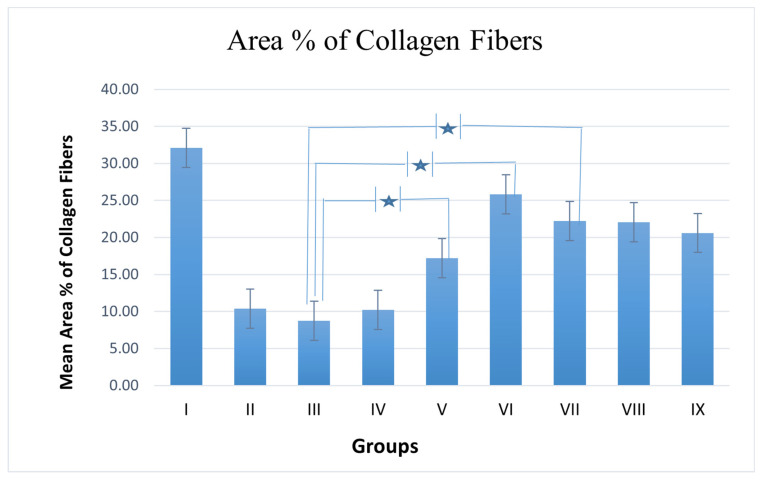
Mean area % of collagen fibers, data presented as mean positive or negative SE, *n* = 6. Star: significant difference.

Microscopical examination for different skin samples demonstrated the following: group I revealed normal histological structures of different skin layers, including an apparent intact thin epidermal layer with intact covering epithelium, as well as an intact dermal layer (Figure 3A,B). It also showed normally distributed collagen fibers (Figure 3A) up to 32.1% of the mean area percentage of the dermal layer content, as shown in Masson’s trichrome-stained tissue sections of all the samples (Figure 4 and Figure 3A,B). The group II samples (burned, non-infected, untreated) showed a wide area of wound gap, covered with scabs from a necrotic tissue depress with a significant loss and necrosis of the underlying dermal layer, replaced with newly formed granulation tissue with abundant inflammatory cell infiltrates, fibroblastic proliferation, as well as many congested subcutaneous blood vessels (BVs) (Figure 3C,D). This group also displayed minimal records of mature collagen fibers (Figure 4B) up to 10.38% of the mean area percentage of the dermal layer content (Figure 5). Group III (burned, infected, untreated) demonstrated almost the same records as the group II samples, with even more severe records of mixed inflammatory cell infiltrates in dermal and subcutaneous tissue, as well as focal dermal hemorrhagic patches (Figure 3E,F). This group also showed more minimal records of mature collagen fibers (Figure 4C), up to 8.75% of the mean area percentage of the dermal layer content (Figure 5). Group IV (burned, infected, treated with control hydrogel) demonstrated almost the same records as group II (Figure 3G,H), also with minimal records of mature collagen fibers (Figure 4D) up to 10.22% of the mean area percentage of the dermal layer content (Figure 5). Group V (burned, infected, treated with Phage 1 hydrogel) showed persistent records of ulcerated wound gap with epidermal loss and necrotic tissue depression. However, highly cellular subepidermal granulation tissues were observed with abundant records of fibroblastic activity, and mixed inflammatory cell infiltrates (Figure 3I,J). The group also showed mildly higher records of dermal mature collagen fibers (Figure 4E) up to 17.2% of the mean area percentage of the dermal layer content (almost twofold more when compared to Group III Control samples) (Figure 5).

The group VI samples (burned, infected, treated with phage 5 hydrogel) showed persistent records of an ulcerated wound gap with epidermal loss and a necrotic tissue depression with moderate persistent records of inflammatory cell infiltrates in deep dermal and subcutaneous layers (Figure 3K,L). However, significant acceleration in dermal mature collagen fiber formation (Figure 4F) up to 25.8% of the mean area percentage of the dermal layer content was shown (almost threefold more when compared to the Group III Control samples) (Figure 5). The group VII samples (burned, infected, treated with phage cocktail hydrogel (vB_Pae_SMP1 + vB_Pae_SMP5) showed a significantly accelerated wound gap closure with a complete re-epithelialization with a thick hyperkeratotic epidermal layer. In addition, there was a significant reduction in inflammatory cell infiltrates with a higher fibroblastic activity (Figure 3M,N) and a moderate maturation of dermal collagen fibers (Figure 4G) up to 22.2% of the mean area percentage of the dermal layer content (almost 2.5-fold higher when compared to Group III Control samples) (Figure 5) just like the positive control group VIII.

However, the group VIII positive control samples (burned, infected, treated with Silverburn^®^) showed almost the same records as the Group VII samples (Figure 3O,P) as well as a moderate maturation in dermal collagen fibers (Figure 4H) up to 22% of the mean area percentage of the dermal layer content (Figure 5). However, group IX, the positive control (burned, infected, treated with Iruxol^®^), showed persistent records of a narrow ulcerated wound gap covered with a scab of a necrotic tissue depress with occasional records of subepidermal hemorrhagic patches and a significant reduction in inflammatory cell infiltrates in dermal and subcutaneous layers (Figure 3Q,R) with a moderate maturation in dermal collagen fibers (Figure 4I) up to 20.6% of the mean area percentage of the dermal layer content (Figure 5).

## 4. Discussion

CRPA has emerged as a serious pathogen on a global scale due to its limited treatment options and, therefore, is placed in the priority 1-critical category of the World Health Organization’s global priority pathogens for research and development of novel antimicrobials [48]. Accordingly, this situation has encouraged a reconsideration of bacteriophage therapy as a possible promising treatment option. In fact, several studies have reported a sporadic rise in the use of antibacterial phage therapy over the course of the past century [49,50,51]. Additionally, there are some advantages that make phage therapy stand out, including the high host specificity, specific reproduction at the site of infection, the eligibility of single or rare administration, and the benefit of being effective against other pan-drug resistant bacteria [52].

Despite studies demonstrating the clinical effectiveness of phages, further research is still needed in this field due to the information gap regarding the in vivo studies. As a result, our study aimed to test the *lytic activity* of two novel isolated bacteriophages formulated as hydrogels to combat CRPA-infected burns using an appropriate skin animal model. This was accomplished by isolating three clinical *P. aeruginosa* from a third-degree burned patient who is experiencing severe complications and rejection in responding to several antimicrobial agents employed in treatment. The antibiogram analysis showed that the respective isolates had a high level of resistance to several antimicrobial agents, including carbapenems, in addition to the MIC of all isolates revealing that they are all carbapenem-resistant and hence classified as CR-XDR isolates [25]. Therefore, these isolates can undoubtedly cause life-threatening conditions for patients as their colonization of a burn wound frequently results in a disseminated infection and occasionally septic shock and mortality. Accordingly, early disclosure of the widespread dissemination of CRPA with devastating effects within clinical settings is vital for the prompt implementation of infection control measures [53].

CRPA, a perturbing carbapenem-resistant pathogen, was selected as the bacterial host for the purpose of isolating phages active against carbapenem-resistant organisms [12,13,53]. Three CRPA isolates were collected from different clinical specimens. They were resistant to all the antibiotics tested, including imipenem and meropenem. The isolation of the bacteriophage was performed by incubating a freshly grown bacterial host with an environmental sample. Then the bacterial cells were removed, and the suspension was purified using centrifugation and chloroform treatment. For this purpose, 15 environmental samples were collected from a hospital sewage source. Only two lysates continued to produce positive results, suggesting sewage was an outstanding source for phage isolation against *P. aeruginosa* [54,55].

Over the course of time, sewage was believed to be an important reservoir of bacteriophages infecting *P. aeruginosa* [54]. The plaque assay offers information on a specific lysate’s purity, as well as the count of viruses [32,56]. A single pure virus is present if it only manifests as one type of plaque with similar morphology, size, and shape, while the presence of multiple viruses is indicated if the plaque assay results in a variety of plaque forms with different characteristics. Additionally, the numbers for identical plaques are inserted into a mathematical formula that roughly predicts the count of viruses that were present in the original lysate in plaque-forming units per ml (PFU/mL) [32,57]. The two phage suspensions had initial titers that were relatively high and reproducible (>10^8^ PFU/mL). Plaque morphology may reveal the phage type. Lytic (virulent) phages typically have clear, transparent plaques, whereas phages with a lysogenic ability (temperate phages) have opaque, turbid ones [58]. Additionally, some phages produce plaques encircled by halos. Halos are attributed to the diffusion of bacteriophage depolymerase enzymes, which are primarily active against bacterial cell walls biofilms [59]. The vB_Pae_SMP1 and vB_Pae_SMP5 plaques were clear, circular, and had regular whole margins. They were also small (2–5 mm) in diameter, which indicated they were lytic [60]. Additionally, the plaques with halos surrounding them were seen to increase in size with increasing the incubation time or after being kept at room temperature for several days, suggesting an anti-biofilm activity. However, this requires additional testing against organisms that produce biofilms [61].

Normally, candidates for phage therapy are chosen using strict criteria, such as being obligate lytic, having a wide host range, and naturally having high stability [62]. As a result, it was important to investigate some of the phages’ primary characteristics. A bacteriophage’s host range is restricted to the genus, species, and strains of bacteria that it can infect [57]. This is undoubtedly essential when using a phage in therapy. As shown in the results, both vB_Pae_SMP1 and vB_Pae_SMP5 lysates were active against two out of the three tested XDR *P. aeruginosa* clinical isolates; however, none of the two lysates showed *lytic activity* against any of the remaining 11 clinically relevant Gram-negative pathogens, which suggested their restricted host range and specificity toward *P. aeruginosa* isolates. Therefore, the two lysates were chosen with isolate PA2 for further studies. A phage’s host range should be constrained to a single species to prevent it from attacking bacteria other than the disease-causing one, protecting the host’s microbiome. Therefore, a phage with a restricted host range is preferred in terms of species. However, a phage that infects many, if not all, strains within this species is advantageous. It suggests that it is suitable for an empirical application, just like broad-spectrum antibiotics [57]. *Furthermore,* the traditional phage isolation technique, which assigns just one host on which phages are supposed to grow, may contribute to their generally restricted host range [57,63,64].

The morphology of phage particles is primarily used for their typical classification. However, the discrepancies among numerous bacteriophage with similar morphology but divergent genomic content has led to recent proposals to cancel the traditional classification of having one bacteriophage order Caudovirales with three major families [65]. Currently, the class Caudoviricetes comprises tailed phage families, most famously the former families Myoviridae, Siphoviridae, and Podoviridae representing the different tailed morphotypes. Phages belonging to these morphotypes share icosahedral capsids with double-stranded (ds) DNA, but their tail shapes vary [66]. Transmission electron microscopy (TEM) examination is the most significant technique for phage visualization [67]. vB_Pae_SMP1 and vB_Pae_SMP5 phage suspensions were introduced for TEM [28]. The morphological features of the vB_Pae_SMP1 and vB_Pae_SMP5 virus particles demonstrated that they seemed to be tailed; hence, they are proposed to be members to class Caudoviricetes. They have an icosahedral head and lack connectors at the head-to-tail junction. However, vB_Pae_SMP5 had an extra base plate with a 35 nm fiber noticed at the end of the tail. These properties excluded the possibility of belonging to the former family Myoviridae [67]. They were marked with a long, flexible non-contractile tail (~170 nm) and an icosahedral head, ~60 nm in diameter. It was suggested that the vB_Pae_SMP1 most likely belong to the siphoviruses based on the morphological similarity to the descriptions presented on the Viral Zone website (“Siphoviridae ViralZone”) and in accordance with the guidelines compiled by the International Committee on the Taxonomy of Viruses (ICTV)—ninth report [68]. Phage vB_Pae_SMP5 was genotypically confirmed to belong to the class Caudoviricetes, genus *Septimatrevirus*, which is one of the newly established genera with no assigned family yet, and we propose that it can be considered family Septimatreviridae. Genomic analysis of vB_Pae_SMP1 is undergoing.

For testing the in vitro effect of the isolated phages formulated as hydrogels on a CRPA-infected burn, CMC was chosen for our hydrogel preparation. Cellulose is a widely available biopolymer with distinct properties, most importantly being that it is very water-soluble and forms superabsorbent hydrogels with excellent mechanical and viscoelastic properties. Cross-linked CMC-based hydrogels have recently been researched as potential dermal drug delivery systems for bacteriophages and antibiotics because of these features [69,70].

The anti-CRPA activity of the tested hydrogel was evaluated using the cup-plate method. The results showed the formation of inhibition zones around both the positive control cups containing either phage vB_Pae_SMP1 or vB_Pae_SMP5 lysates and around the cups containing either the tested hydrogel of phage vB_Pae_SMP1 or vB_Pae_SMP5 while the negative control cups containing the CMC- hydrogel alone exhibited no inhibition zones indicating the benefit of CMC hydrogel as a vehicle for delivering the bacteriophages vB_Pae_SMP1 and vB_Pae_SMP5 in a sustained manner [71].

To evaluate the ability of the bacteriophages formulated as hydrogels to alleviate the pathogenicity of such CRPA isolate (PA2), a thermal injury model was designed in rats infected with the CRPA isolate (PA2). Numerous studies in the literature applying superficial skin infection in mice/rats have been reported [18,72,73]. The prepared bacteriophage-hydrogel was applied to the infected burn following the establishment of the infection. The calculated survival rates for the treated groups were compared to those of other control groups. Histopathological examination and the percentage of dermal collagen fibers of the burned infected rat skin were determined as well [18]. Our study’s findings showed that the tested hydrogel containing the bacteriophages increased the survival rate, as all the animals given this tested hydrogel survived. These findings come in accordance with previous studies, which reported a higher survival rate after phage treatment compared to the control [74,75,76]. This could be attributed to the beneficial topical use of the phages in reducing the number of bacteria in the wound and reducing their dissemination at the end of phage treatment, as previously reported [77]. Additionally, the results revealed that rats treated with control hydrogel (the vehicle) without the bacteriophages had higher survival rates than the untreated rats. This could be due to the moisturizing properties of the hydrogel and its function as a physical barrier that shields the wound, as was mentioned in a previous study that discussed the benefits of hydrogels in wound care [78].

The histological examination of various wounded groups showed different degrees of tissue damage and healing processes. To ensure the reliability of the results and relate the healing activity entirely to bacteriophage, an additional control group (group IV), aside from the untreated group, was designed for our study. In this control group (Group IV), a hydrogel made solely from the vehicle without bacteriophage was topically applied to the infected burn, and the results were evaluated. In a similar manner, group II, which had non-infected burns, was utilized to assess the damage produced by XDR *P. aeruginosa* infection in the other groups relative to this group. Moreover, another control group (group III) was designed in which XDR *P. aeruginosa* infection was induced to the burn and left untreated. This group was established to investigate the virulence and the properties of *P. aeruginosa* pathogenicity, assess its persistent inflammation that delays healing and boosts antimicrobial tolerance, and the efficacy of topical bacteriophages in the burn-wound infection model, as previously reported [18,79]. Two groups were designed as positive controls to be used as references for comparing antibacterial activity and wound healing properties. In one positive control group (group VIII), the infected burn was treated with silver sulfadiazine, which is well-known for its antibacterial activity. Silver sulfadiazine is the topical treatment of choice in severe burns and is used almost globally today in preference to compounds such as silver nitrate and mafenide acetate [80]. In the other positive control group (group IX), the infected burn was treated with collagenase due to its wound-healing properties.

Collagenase, a Clostridium histolyticum-derived enzyme debriding agent, is utilized in clinical practice to treat infected and surgical wounds [81]. Previous research has found that collagenase cleans the necrotic tissue of the wound using an enzymatic technique, accelerates the formation of granulation tissue and subsequent re-epithelialization, and increases the activation of fibroblasts, myofibroblasts, and collagen in rat dermal wounds [80]. Therefore, silver sulfadiazine and collagenase were used in our study as positive controls for the treatment of rat skin wounds, as reported in several studies [80,82,83]. Our results revealed that topical application of bacteriophage hydrogel in groups (V, VI, VII) showed a significant difference (*p* < 0.05) in suppressing local wound infection and promoting skin regeneration, as well as higher records and significant acceleration of dermal mature collagen fiber formation compared to the control group III (burned, infected, untreated) indicating wound healing process. Thus, our results confirmed that phages could be promising in preventing wound-associated infections with *P. aeruginosa* [84]. Our findings were in accordance with the study conducted by Mendes et al., which proved an epithelial gap reduction in wounds treated by phages infected by *P. aeruginosa* and, therefore, was proof that phages can enhance wound healing as well as combat the infection [85].

Several studies have reported that a topically administered bacteriophage treatment may be effective in resolving and healing chronic wound infections in animal models [86,87,88]. Our findings revealed that the group treated with the phage cocktail (Group VII) showed more significant accelerated wound gap closure with complete re-epithelialization. The treatments also resulted in a significant reduction (*p* < 0.05) in inflammatory cell infiltrates with higher fibroblastic activity when compared to the other single phage-treated groups (Group V and Group VI). This could suggest the synergistic activity of using the phage cocktail compared to using a single phage alone. Phage cocktails have the potential to target a wider variety of strain-specific microorganisms, and their use is considered a crucial strategy for limiting the development of bacterial resistance [84]. In fact, Pinto and his colleagues reported that after just a few hours of monophage therapy, several resistant bacterial strains had emerged. When compared to monophage therapy, phage cocktails accelerate the rate of bacterial death and decrease the number of phage-resistant bacterial mutants [89]. Additionally, a study performed on mice revealed that phage cocktails led to a significant decrease in wound bioburden, faster wound closure, and greater wound contraction. Furthermore, in vitro stability studies and in vivo phage titer determination have shown a correlation between better phage persistence at the wound site and liposomal entrapment of the phage cocktail [90].

## 5. Conclusions

In this, study two lytic phages of the class Caudoviricetes, named VB_Pae_SMP1 and VB_Pae_SMP5, were isolated from sewage samples, purified and charcaterized. The lytic activities of the respective phages either each alone or as cocktail were evlauted both in vitro and in vivo using XDR *Pseudomonas aeruginosa*-infected skin thermal injury rat model. The phae VB_Pae_SMP5 was genotypically confirmed to beloged the class Caudoviricetes; genus Septimatrevirus. Each of the phage lysate or the two-phage cocktail formulated as a hydrogel for topical treatment has the power to manage the infection of XDR CRPA in burn wounds and decrease the mortality rate of the tested rats. Additionally, it encourages healing at the injury site, as evidenced by the histopathological examination where restored epithelium and activated fibroblasts were noticed. The two phages formulated hydrogels improved wound healing as well as mortality rates of the tested animals. Therefore, each of the phage lysate or the two-phage cocktail formulated as a hydrogel is a promising topical formula for future clinical use in burn wounds caused by severe CRPA infections. However, further clinical studies should be done to ensure suitability of the respective phage hydrogels for clinical application in humans.

## Figures and Tables

**Table 1:** Summary for susceptibility pattern, MIC, phenotypic and genotypic detection of CPs *P. aeruginosa* isolates.

Isolate Code	Susceptibility Pattern						MIC of IMP (μg /mL)	Phenotypic Tests			Carbapenemase Genes
	AK	AT	CIP	LEV	IMP	MER		CDT	mCIM	BCT	
CRPA1	R	R	R	R	R	R	>1024	−	−	+	*bla*KPC
CRPA2	R	R	R	R	R	R	>512	−	+	+	*bla*OXA-48
CRPA3	R	R	R	R	R	R	32	−	+	+	*bla*OXA-48

Ak, amikacin; AT, aztreonam; CIP, ciprofloxacin; LEV, levofloxacin, IMP, imipenem; MER, meropenem; MIC, minimum inhibitory concentration; blaKPC, gene coded for Klebsiella pneumoniae carbapenemase (group A beta-lactamase); blaOXA-48, gene coded for oxacillinase (group D Beta-lactamase), modified carbapenem inactivation method (mCIM), combined disk test (CDT), and blue-Carba test (BCT).

**Table 2:** Host range of the lytic properties of phages vB_Pae_SMP1 and vB_Pae_SMP5.

Isolate Code	Microorganism	Spot Test	
		Lysate vB_Pae_SMP1	Lysate vB_Pae_SMP5
CRPA1	*P. aeruginosa*	−	+
CRPA2	*P. aeruginosa*	+	+
CRPA3	*P. aeruginosa*	+	−

**Table 3:** Genomic and phenotypic characterization of the phage vB_Pae_SMP5.

Parameters	Phage vB_Pae_SMP5
Molecular type	Genomic DNA
Genomic size (bp)	43070 bp
Proteins/ORFs	57 (46 coded by + frames and 11 coded by - frames)
Isolation Source	sewage
Host	XDR *Pseudomonas aeruginosa* clinical isolates CRPA1 and CRPA2
Class	Caudoviricetes
Family	Currently unassigned (formerly Siphoviridae, suggested Septimatreviridae)
Genus	*Septimatrevirus*
Species	unclassified

**Table 4:** Percentage of Survival rate of the examined rats.

Group	Description	Rats Survival %
I	Normal Control, intact, non-infected, untreated	100
II	Control, Burned, non-infected, untreated	80
III	Control, burned, infected, untreated	40
IV	Control, Burned, infected, treated with control hydrogel	60
V	Burned, infected, treated with Phage 1 hydrogel	100
VI	Burned, infected, treated with phage 5 hydrogel	100
VII	burned, infected, treated with phage cocktail hydrogel (vB_Pae_SMP1 + vB_Pae_SMP5)	100
VIII	Positive Control, burned, infected, treated with Silverburn^®^	100
IX	Positive Control, burned, infected, treated with Iruxol^®^	100

## Data Availability

The data supporting reported results are found in the manuscript and supplementary file. The genomic sequence of phage vB_Pae_SMP5 has been deposited in the NIH-funded BV-BRC database, and the genome will be publicly released upon manuscript publication. The BV-BRC accession is 2731619.92, and its link is https://www.bv-brc.org/view/Genome/2731619.92. (accessed on 7 December 2022).

## Supplementary Materials

The following supporting information can be downloaded at: https://www.mdpi.com/article/10.3390/v14122760/s1, Figure S1: Plaque assay results of vB_Pae_SMP5 at different dilutions; Figure S2: Plaque morphology as presented by phages vB_Pae_SMP1 and vB_Pae_SMP5. Plaques are clear, regularly circular, small in size (diameter of 2–5 mm), and halos are shown around the plaques; Figure S3: Spot test of phages vB_Pae_SMP1 and vB_Pae_SMP5 against *Pseudomonas aeruginosa* isolate showing clear spots and proving their lytic abilities; Figure S4: In vitro anti-CRPA activity of the tested hydrogels. Phage vB_Pae_SMP1 and vB_Pae_SMP5: Positive control. vB_Pae_SMP1 +CMC, vB_Pae_SMP5 +CMC: Tested hydrogels, C: negative control; Table S1: Genomic analysis (resulted contigs and putative functions of the resulted open reading frames) of the phage vB_Pae_SMP5.
