# Supplementary material for: In Vitro and Pre-Clinical Evaluation of Locally Isolated Phages, vB_Pae_SMP1 and vB_Pae_SMP5, Formulated as Hydrogels against Carbapenem-Resistant Pseudomonas aeruginosa
Source: Viruses. 2022 Dec 11;14(12):2760. doi: 10.3390/v14122760 (PMC9780878; doi:10.3390/v14122760)
Supplement: Supplementary file 1 [file viruses-14-02760-s001.zip › viruses-2057384-supplementary.pdf]

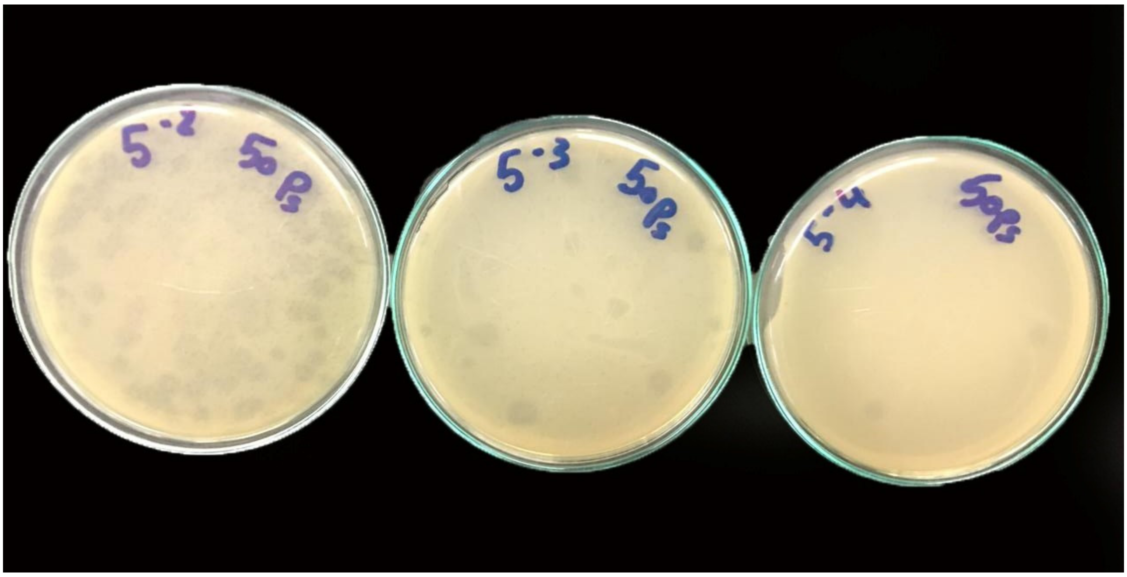

Figure S1. Plaque assay results of VB_PAE_SMP5 at different dilutions.

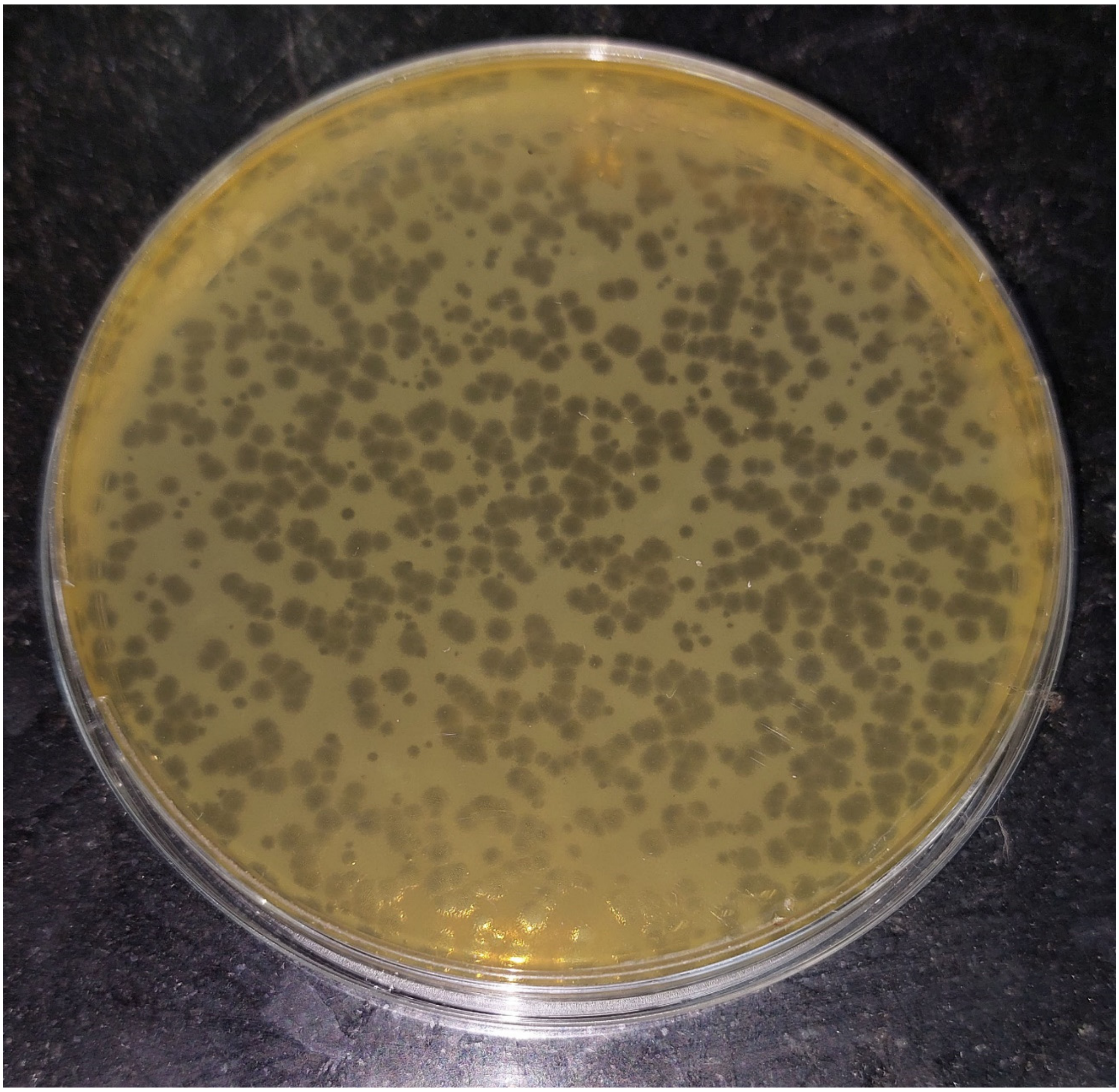

Figure S2. Plaque morphology as presented by phages VB_PAE_SMP1 and VB_PAE_SMP5 . Plaques are clear, regularly circular, small in size (diameter of 2-5 mm) and halos are shown around the plaques.

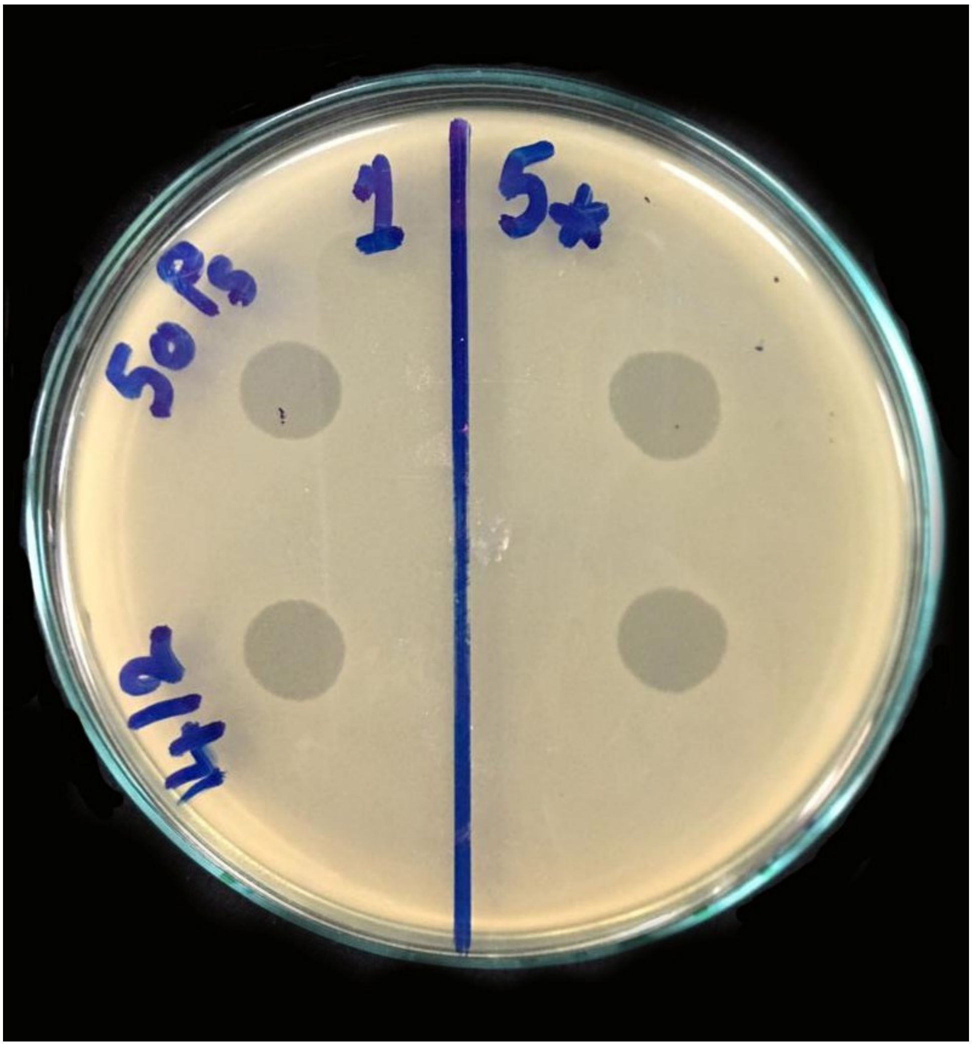

Figure S3. Spot test of phages VB_PAE_SMP1 and VB_PAE_SMP5 against *Pseudomonas aeruginosa* isolate showing clear spots and proving their lytic abilities.

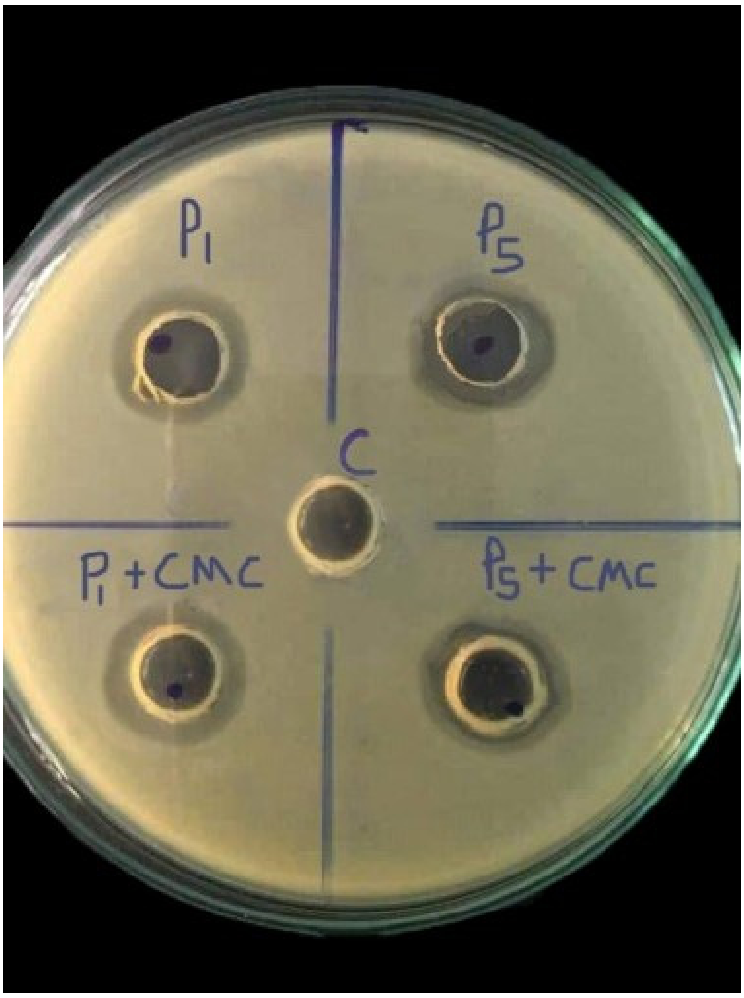

Figure S4. In vitro anti-CRPA activity of the tested hydrogels. Phage VB_PAE_SMP1 and VB_PAE_SMP5 : Positive control. VB_PAE_SMP1 +CMC, VB_PAE_SMP5 +CMC: Tested hydrogels, C: negative control.

Table S1. Genomic analysis (resulted contigs and putative functions of the resulted open reading frames) of the phage vB_Pae_SMP5.

Feature_id	Location	Start	Stop	Strand	Curated function	RASTtk annotation	BLASTP best hit
fig|2731619.91.peg.1	48_536	48	536	+	Phage terminase, small subunit	Phage protein	possible small terminase subunit
fig|2731619.91.peg.2	517_2103	517	2103	+	Phage terminase, large subunit	Phage terminase, large subunit	terminase-like family protein / large terminase subunit
fig|2731619.91.peg.3	2117_3622	2117	3622	+	Phage portal protein	Putative structural protein (ACLAME 256)	portal protein
fig|2731619.91.peg.4	3634_4728	3634	4728	+	Phage minor capsid protein	Phage minor capsid protein	F-like head morphogenesis protein
fig|2731619.91.peg.5	4765_5484	4765	5484	+	Phage scaffold protein	Phage protein (ACLAME 257)	scaffold protein
fig|2731619.91.peg.6	5487_6464	5487	6464	+	Phage major capsid protein	Phage portal or tail protein (ACLAME 11)	major capsid protein
fig|2731619.91.peg.7	6534_6938	6534	6938	+	Phage protein	Phage protein	hypothetical protein ORF011
fig|2731619.91.peg.8	7004_7375	7004	7375	+	Phage virion structural protein	Phage protein	virion structural protein
fig|2731619.91.peg.9	7388_7906	7388	7906	+	Phage head-to-tail connector	Phage protein (ACLAME 313)	virion protein: head-to-tail connector complex protein
fig|2731619.91.peg.10	7910_8290	7910	8290	+	Phage head-tail joining protein	Phage protein (ACLAME 302)	Head-tail joining protein
fig|2731619.91.peg.11	8287_8742	8287	8742	+	Phage minor tail protein	Peptidoglycan binding protein	Minor tail protein
fig|2731619.91.peg.12	8903_10345	8903	10345	+	Phage major tail tube protein	Phage protein	major tail tube protein
fig|2731619.91.peg.13	10409_10837	10409	10837	+	Phage tail chaperonin	Phage protein	tail chaperonin
fig|2731619.91.peg.14	10846_11202	10846	11202	+	Phage tail chaperonin	Phage protein	tail chaperonin
fig|2731619.91.peg.15	11171_11605	11171	11605	+	Phage tail completion protein	Phage protein (ACLAME 188)	tail completion protein
fig|2731619.91.peg.16	11611_15225	11611	15225	+	Phage tail tape measure protein	Phage tail tape measure protein	tape measure protein
fig|2731619.91.peg.17	15226_16200	15226	16200	+	Phage structural protein	Phage protein (ACLAME 678)	virion protein
fig|2731619.91.peg.18	16200_17144	16200	17144	+	Phage tail assembly protein	Phage protein (ACLAME 761)	virion structural protein / tail assembly protein
fig|2731619.91.peg.19	17150_18862	17150	18862	+	Phage tail assembly structural protein	Phage protein (ACLAME 463)	virion structural protein/ tail assembly structural protein
fig|2731619.91.peg.20	18862_19686	18862	19686	+	FAD/FMN-containing dehydrogenase	Gene Transfer Agent (GTA) ORF13 or FAD/ FMN-containing dehydrogenase (ACLAME 176)	FAD/FMN-containing dehydrogenase
fig|2731619.91.peg.21	19690_22131	19690	22131	+	Phage central tail hub protein	Phage protein (ACLAME 455)	central tail hub

fig|2731619.91.peg.22	24183_22132	24183	22132	-	DNA polymerase B region	DNA polymerase B region	DNA polymerase
fig|2731619.91.peg.23	25337_24195	25337	24195	-	Phage replicative clamp	Phage protein	replicative clamp
fig|2731619.91.peg.24	25548_25321	25548	25321	-	Phage protein	Phage protein	hypothetical protein vB_Pae_PS9N_00041
fig|2731619.91.peg.25	27208_25553	27208	25553	-	Phage DNA helicase	Phage DNA helicase (ACLAME 43)	putative helicase/ DEAD box helicase
fig|2731619.91.peg.26	28097_27201	28097	27201	-	Phage exonuclease RecB	Phage protein	exonuclease/ RecB exonuclease
fig|2731619.91.peg.27	28731_28204	28731	28204	-	hypothetical protein	hypothetical protein	hypothetical protein TehO_047
fig|2731619.91.peg.28	29556_28810	29556	28810	-	Phage single-stranded DNA-binding protein	Phage protein (ACLAME 618)	hypothetical protein 40_00002/ single-stranded DNA-binding protein
fig|2731619.91.peg.29	30331_29615	30331	29615	-	Phage recombinase RecA	Phage methyl-accepting chemotaxis protein (ACLAME 1366)	RecA/ AAA domain-containing protein
fig|2731619.91.peg.30	30826_30386	30826	30386	-	Phage protein	Phage protein	hypothetical protein ORF033
fig|2731619.91.peg.31	31460_30903	31460	30903	-	Phage MazG nucleotide pyrophosphatase/toxin-antitoxin	Phage MazG nucleotide pyrophosphatase/toxin-antitoxin (ACLAME 361)	MazG
fig|2731619.91.peg.32	31598_31786	31598	31786	+	Phage putative transcriptional regulator	Phage protein	hypothetical protein ORF035/ transcriptional regulator
fig|2731619.91.peg.33	31776_34097	31776	34097	+	Phage DNA primase/helicase	DNA primase/helicase, phage-associated	DNA primase/helicase
fig|2731619.91.peg.34	34249_34518	34249	34518	+	hypothetical protein	hypothetical protein	hypothetical protein KAK25_00045
fig|2731619.91.peg.35	35042_34614	35042	34614	-	hypothetical protein	hypothetical protein	hypothetical protein KAK25_00040
fig|2731619.91.peg.36	35065_35283	35065	35283	+	hypothetical protein	hypothetical protein	hypothetical protein
fig|2731619.91.peg.37	35280_35537	35280	35537	+	hypothetical protein	hypothetical protein	hypothetical protein S3_044
fig|2731619.91.peg.38	35858_36133	35858	36133	+	hypothetical protein	hypothetical protein	hypothetical protein KAK25_00042
fig|2731619.91.peg.39	36130_36309	36130	36309	+	hypothetical protein	hypothetical protein	hypothetical protein KAK25_00044
fig|2731619.91.peg.40	36612_36833	36612	36833	+	hypothetical protein	hypothetical protein	hypothetical protein
fig|2731619.91.peg.41	36882_37256	36882	37256	+	Phage protein	Phage protein	hypothetical protein vBPaeSC1_02
fig|2731619.91.peg.42	37317_37538	37317	37538	+	Phage RNA polymerase-binding protein	hypothetical protein	hypothetical protein KAK25_00047 / RNA polymerase-binding protein
fig|2731619.91.peg.43	37535_38071	37535	38071	+	Phage endonuclease	Phage protein (ACLAME 766)	endonuclease

fig|2731619.91.peg.44	38071_38274	38071	38274	+	hypothetical protein	hypothetical protein	hypothetical protein KAK25_00049
fig|2731619.91.peg.45	38271_38450	38271	38450	+	hypothetical protein	hypothetical protein	hypothetical protein
fig|2731619.91.peg.46	38511_38816	38511	38816	+	Phage protein	Phage protein	hypothetical protein
fig|2731619.91.peg.47	38833_39123	38833	39123	+	Phage endolysin	Phage endolysin	hypothetical protein S4_053
fig|2731619.91.peg.48	39116_39349	39116	39349	+	Phage protein	Phage protein	hypothetical protein KAK25_00053
fig|2731619.91.peg.49	39420_39587	39420	39587	+	Phage protein	Phage protein	hypothetical protein vBPaeW3_33
fig|2731619.91.peg.50	39578_40045	39578	40045	+	Putative dCMP deaminase	Putative dCMP deaminase (ACLAME 374)	dCMP deaminase
fig|2731619.91.peg.51	40051_40434	40051	40434	+	Phage protein	Phage protein	hypothetical protein ORF050
fig|2731619.91.peg.52	40469_40678	40469	40678	+	Phage protein	Phage protein	hypothetical protein HL17_gp51
fig|2731619.91.peg.53	40762_41334	40762	41334	+	Phage protein	Phage protein	hypothetical protein ORF052
fig|2731619.91.peg.54	41483_41737	41483	41737	+	Phage protein found in lysis cassettes	Phage protein	hypothetical protein AVU20_gp01/ member of phage protein family found in lysis cassettes
fig|2731619.91.peg.55	41734_42000	41734	42000	+	Phage holin	Phage protein	holin
fig|2731619.91.peg.56	41993_42538	41993	42538	+	Phage tail fiber-associated endolysin	Phage tail fiber protein (long tail fiber)	endolysin / TPA: Tail associated lysozyme
fig|2731619.91.peg.57	42550_42855	42550	42855	+	Phage Rz protein	Phage protein	Rz protein
